# Combining Telephone Surveys and Fishing Catches Self-Report: The French Sea Bass Recreational Fishery Assessment

**DOI:** 10.1371/journal.pone.0087271

**Published:** 2014-01-29

**Authors:** Delphine Rocklin, Harold Levrel, Mickaël Drogou, Johanna Herfaut, Gérard Veron

**Affiliations:** 1 UMR M101, Aménagement des Usages, des Ressources et des Espaces marins et littoraux, Observatoire des Sciences de l'Univers – Institut Universitaire Européen de la Mer, Université de Brest, Brest, France; 2 UMR M101, Aménagement des Usages, des Ressources et des Espaces marins et littoraux, Unité d'Economie Maritime, Ifremer, Plouzané, France; 3 Département des Ressources Biologiques et Environnement, Unité Sciences et Techniques Halieutiques, Ifremer, Plouzané, France; Aristotle University of Thessaloniki, Greece

## Abstract

Fisheries statistics are known to be underestimated, since they are mainly based on information about commercial fisheries. However, various types of fishing activities exist and evaluating them is necessary for implementing effective management plans. This paper assesses the characteristics and catches of the French European sea bass recreational fishery along the Atlantic coasts, through the combination of large-scale telephone surveys and fishing diaries study. Our results demonstrated that half of the total catches (mainly small fish) were released at sea and that the mean length of a kept sea bass was 46.6 cm. We highlighted different patterns of fishing methods and type of gear used. Catches from boats were greater than from the shore, both in abundance and biomass, considering mean values per fishing trip as well as CPUE. Spearfishers caught the highest biomass of sea bass per fishing trip, but the fishing rod with lure was the most effective type of gear in terms of CPUE. Longlines had the highest CPUE value in abundance but not in biomass: they caught numerous but small sea bass. Handlines were less effective, catching few sea bass in both abundance and biomass. We estimated that the annual total recreational sea bass catches was 3,173 tonnes of which 2,345 tonnes were kept. Since the annual commercial catches landings were evaluated at 5,160 tonnes, recreational landings represent 30% of the total fishing catches on the Atlantic coasts of France. Using fishers' self-reports was a valuable way to obtain new information on data-poor fisheries. Our results underline the importance of evaluating recreational fishing as a part of the total amount of fisheries catches. More studies are critically needed to assess overall fish resources caught in order to develop effective fishery management tools.

## Introduction

Marine waters support intensive fishing activities. The total worldwide marine fish production has been estimated as increasing steadily from 16.7 million tonnes in the mid-20th century to about 90 million tonnes at present [1]. However, these figures, mainly based on official fishery landings statistics, are incomplete [2]. They generally do not take into account discarded by-catch, illegally landed catches of the commercial fisheries and unregulated high-seas fisheries [3], [4]. Moreover, they often ignore commercial small-scale artisanal fisheries catches, as well as non-commercial catches such as recreational, sport and subsistence fishing ones [5], which are still under-evaluated. But to manage marine resources responsibly, trustworthy data for all components of fishing activities are necessary.

**Figure 1:**
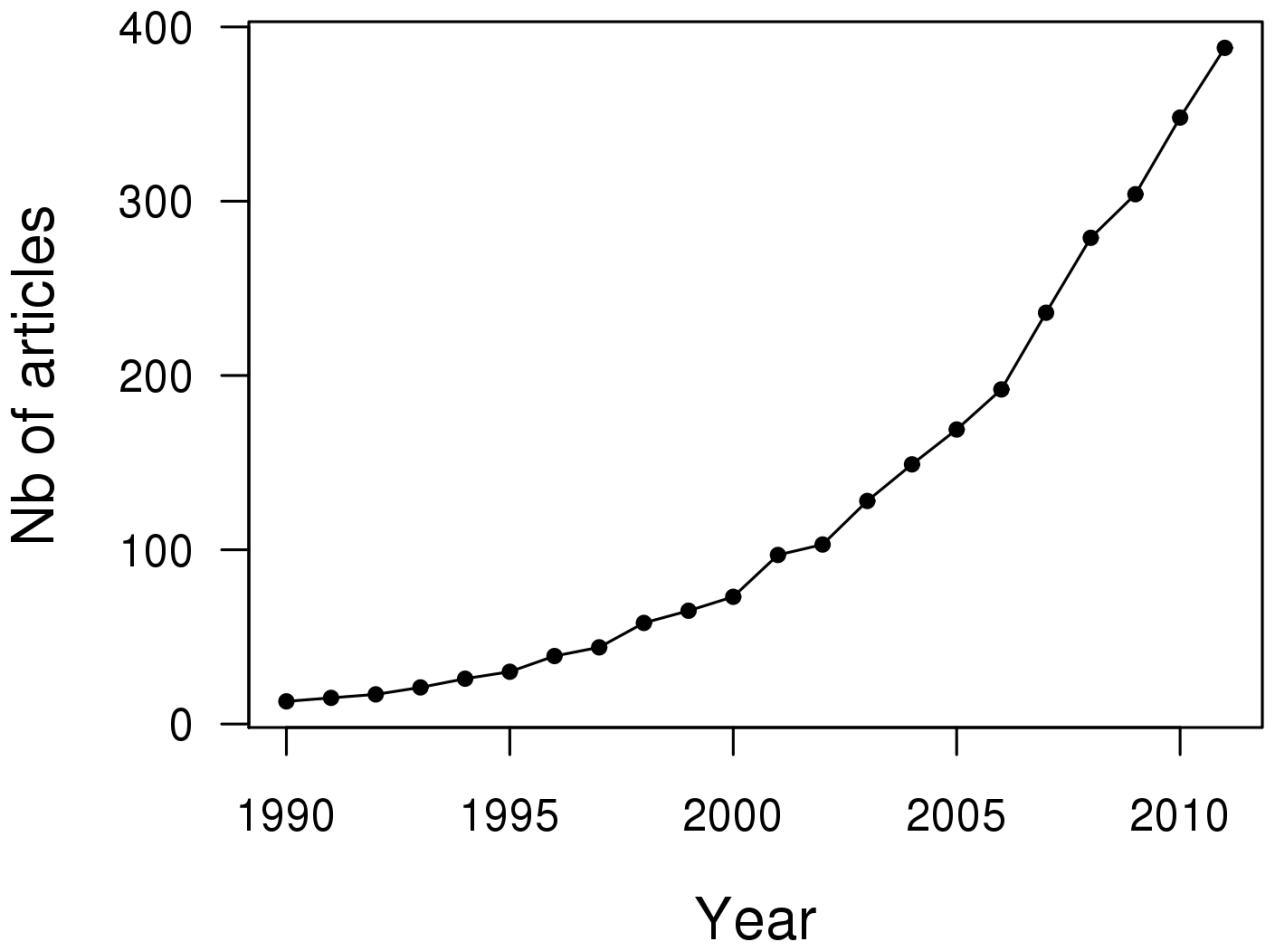
Number of scientific papers citing the term “recreational fisheries” from 1990 until now. Data derived from the research platform “Web of Knowledge” (www.webofknowledge.org).

Recreational fisheries recently appeared to be more important than was once thought, and their potential role in marine resources overexploitation has been pointed out [6], [7]. The number of “recreational fisheries” citations in the research platform “Web of Knowledge” (www.webofknowledge.org) has indeed exponentially increased since 1990 (Fig. 1), demonstrating the quite recent interest in this subject and awareness of its relevance.

The earliest assessments regarded recreational fishing as of low importance. Indeed, in 1994, the National Research Council estimated that US recreational fisheries accounted for about 2% of total marine landings [8]. More recent studies, improving the assessment methodologies, indicated that this percentage ought to be revised upward. It has been estimated that recreational fisheries may account for a percentage of about 10% to 64% of the global fisheries production [6], [9], depending on the considered areas and species [7]. Moreover, in some cases, recreational fishing catches can exceed that of commercial fishing [10]. Based on an extrapolation from the patterns of Canadian recreational fishing onto a global scale, Cooke and Cowx [6] estimated that the total recreational fishing catches may be as much as 47.1 billion fish, of which two-thirds are released, with a retained amount estimated at about 10.86 million metric tonnes. However, these estimates were made using highly general assumptions without taking into account each country's characteristics such as the environmental configuration (freshwater surface area, marine coastline), local policies and/or recreational fishing traditions.

Estimating the characteristics and catches of recreational fisheries thus remains a challenge. Unlike commercial fishing, carried out for profit, and subsistence fishing, carried out for food, recreational fishing is primarily a leisure activity [11] performed in both freshwater and marine environments. It is characterized by a large number of involved fishermen, by the great diversity of participants, the varied timing and frequency of fishing trips, the variability of used gears and access points and the lack of systematic recording of the catches [12], [13]. It is spatially and temporally heterogeneous and there is no uniform method for valuing them [14]. Moreover, the licensing system is not systematic, leading to an overall miscalculation of the population of recreational fishers. All these features make the characteristics and catches of recreational fisheries hard to estimate.

Various methods have been developed for estimating recreational fishing [15]; these generally fall into two parts. The first part focuses on fishing effort: this can be calculated using registries (when a license system exists), aerial flights over geographically limited areas, or telephone surveys for large-scale studies. The second part focuses on evaluating catch rates and species composition: it can be calculated using on-site intercept surveys based on interviews with recreational fishers at the end of their fishing trips, or can draw on a group of fishers, called a panel, declaring their daily catches through diaries or web-based declaration. For example, the US Marine Recreational Information Program (MRIP) [16], formerly called MRFSS (Marine Recreational Fisheries Statistics Survey), was created in 1970 by the National Marine Fisheries Service (NMFS) of the NOAA. It is a large-scale information system which developed various monitoring methods regarding recreational fishing efforts and catches nationwide, through telephone surveys, access point surveys, web-based declaration and mail surveys [5], [17]. Other methods have also been used, such as low-altitude flights for counting fishing boats and shore anglers, thus evaluating the fishing effort and areas, combined with on-site interviews for evaluating catches [18]–[23].

Numerous species, including some viewed as emblematic and/or threatened, are targeted by recreational fishers. Through the European Union Data Collection Framework (DCF), the European Commission has recently required the quantification of the recreational catches of some important European species targeted by both commercial and recreational fisheries: cod (*Gadus morhua*), bluefin tuna (*Thunnus thynnus*), eel (*Anguilla anguilla*), Atlantic salmon (*Salmo salar*) and European sea bass (*Dicentrarchus labrax*) (EC Regulations 199/2008 of 25 February 2008 and EC Decision 2008/949/EC of 6 November 2008). In France, an estimate of the recreational catches of the European sea bass (to be referred to here as sea bass) has been requested for the North Atlantic V-XIV ICES divisions [24]. By the late 1990s, it was already known that recreational fishing for sea bass was popular in England, Wales, the Channel Islands, Ireland, France (especially Brittany), Spain, Portugal and Italy [12]. This species is still highly attractive both on the French Atlantic coasts and in the Mediterranean, and is targeted both by commercial and recreational fishers [25]–[27]. In France, the sea bass is one of the most economically valuable species [12], [28], although it is dependent on the considered métier and season, as well as on the condition of the fish and landed quantities [29]. However, the mean first sale price constantly increased between 1972 and 1984 [30]. Its commercial catches as well as its consumption also raised from 1998 to 2005, significantly contributing to the French market [31]. Nevertheless, although the commercial catches of sea bass in the French Atlantic are well known, no study evaluated the importance of its recreational part.

Our study aimed to enhance the still rare and sparse information on the French sea bass recreational fisheries. Increasing our knowledge of this subject is more than ever a crucial issue since overexploitation is threatening the availability of marine living resources, and managing coastal human activities and natural resources requires a well understanding of their interactions.

This study thus addressed three questions: How can we develop a robust large-scale monitoring system using fishing diaries? What are the main characteristics of the French recreational sea bass fishery? How important are recreational sea bass catches in France?

## Materials and Methods

### Ethics statement

All the participants of this study, only performed in mainland France, were called and interviewed by the BVA polling institute. The data collection and analyses, involving humans categorized by age, gender and other socially constructed groupings, were subjected to a declaration to the CNIL (Commission Nationale de l'Informatique et des Libertés – The French Data Protection Authority), in accordance with the decree n°78-17 of 6 January 1978 on Data Processing, Data Files and Individual Liberties, under the document number 1394854. The BVA polling institute obtained the 3 November 2009 from the CNIL the permanent authorization for performing such studies. The answer of all the called households, whether they consented or not to participate to the study, was recorded into a response file document: the variable “Acceptance” was filled up with “yes” or “no”, and only the participants who provided their verbal consent were interviewed. During the call, the objectives and the proceedings of the study were initially described. Then, the first question was: “Do you agree to answer our questions?”. If they agreed, the telephone interview was conducted. Finally, if they were sea bass recreational fishers, they were asked about their interest in participating as a volunteer for the panel. The whole study was based on the volunteer participation of sea bass recreational fishermen. The volunteers were contacted every three months to verify the smooth proceedings of their data collection and if they still wanted to continue the study. All the volunteers had the direct phone number of the scientist in charge of the study and were encouraged to contact him when necessary. All of the collected data was anonymously analyzed. At the end of the study, a thank you letter and a document summarizing the main results were sent by mail to each volunteer.

### Sampling design

Estimating the number of marine recreational fishers in France is a challenge. This activity is free and no licensing system exists at this time. We focused this first study on the French littoral of the Bay of Biscay, English Channel and North Sea (VIIIa, VIIIb, VIIe, VIIh and VIId ICES areas). In accordance with previous work on recreational fishery surveys [32]–[34], we adopted a dual method combining two large-scale telephone surveys with a fishing diary survey, where the fishing diaries were filled by the recreational fishers themselves.

For the two telephone surveys, the phone numbers were randomly selected in an existing database constituted by the landline and cell phone numbers of French telephone subscribers appearing in the national telephone directory. The sampling design used to contact each household was based on the principle that it should offer to each of them the same and non-negative probability to be contacted. For not inducing bias in the representativeness of the sample, not only the households easily reachable (those often present at their house) were interviewed. Thus, a strict and rigorous call process was used, following these assumptions: (1) the call numbers appeared in a random order (no pool); (2) the calls were done with the same insistence; and (3) the call hours permitted to contact the whole population. Each phone number was therefore called at different hours and days until it was reached or was definitely abandoned after 12 unsuccessful calls.

**Figure 2:**
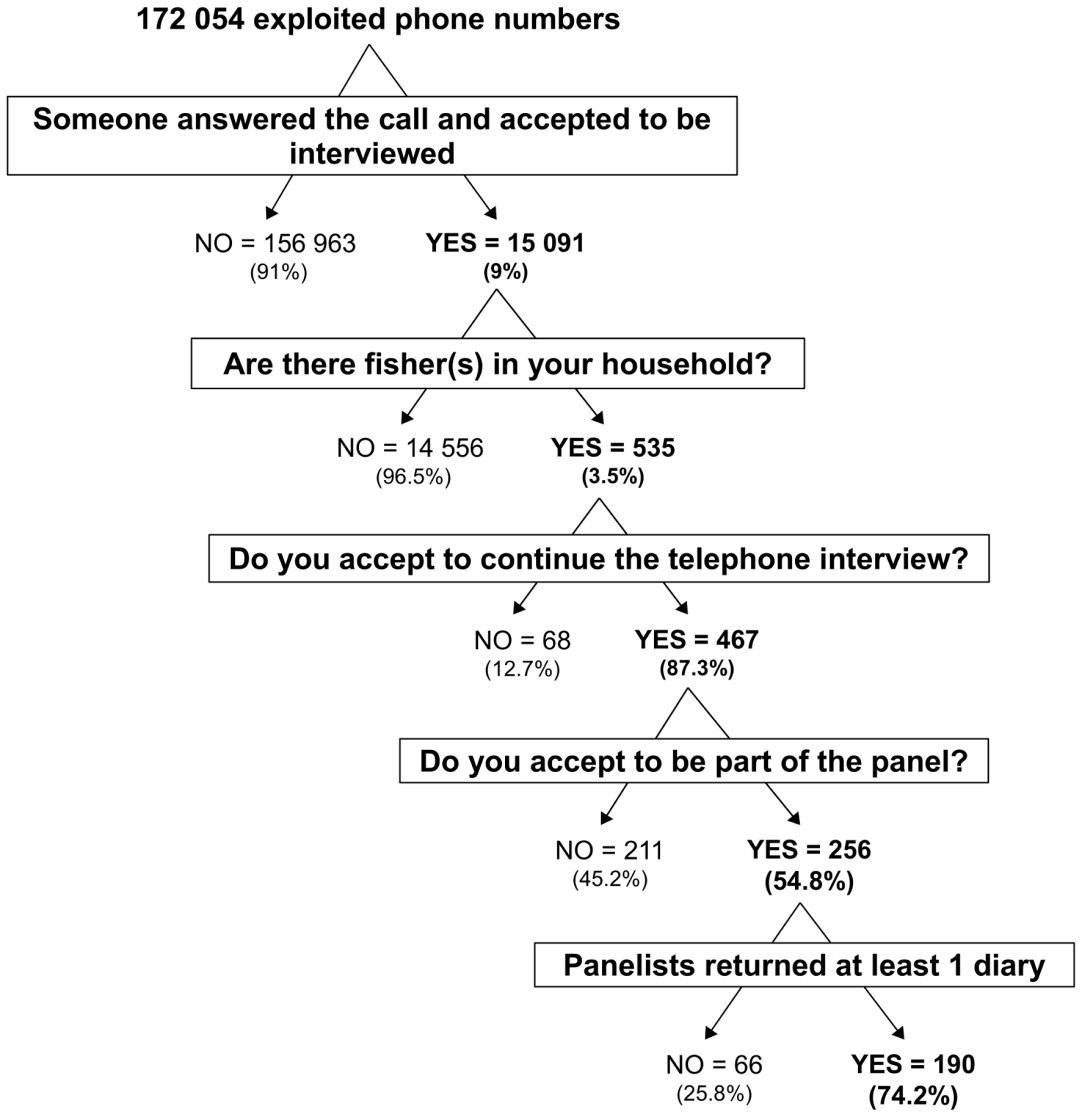
The sea bass-specific survey: followed sampling design.

A first telephone survey, sea bass-specific, was conducted in June and November 2009 in the French coastal departments of the considered study. 172,054 telephone numbers were exploited to obtain a representative sample of 15,091 interviewed households (9%) (Fig. 2). The 91% non-interviewed households corresponded, among others, to an occupied number (37%), a clear refusal to answer the interview (29%), or the non-response to the call (10%). At least one sea bass recreational fisher (here by definition a fisher targeting the sea bass and who has caught at least one sea bass during the last 12 months) was present in 535 (3.5%) of the 15,091 households successfully interrogated. These sea bass fishers were asked if they would agree to continue the interview and 467 of them did so (87.3%). The interview permitted us to collect socio-professional information on recreational fishers targeting sea bass during their fishing trips, as well as general information on their fishing activity during the previous year. The main questions were: What was the main type of gear you used? Did you fish from the shore or from a boat? In which area(s) did you go fishing? When did you go fishing? Approximately how many sea bass and other species did you catch during the last 12 months? Did you release any of these catches? This survey made it possible to identify and precisely describe the main characteristics and practices of recreational sea bass fishers.

At the end of this telephone survey, sea bass fishers were asked to join a panel, that is a group of recreational fishers who agreed to voluntary fill in a fishing diary to report their catches information, during one year. 256 (54.8%) of the 467 sea bass fishers agreed to join the panel. We sent the fishing diaries to the volunteers (one every three months or every 20 fishing trips), as well as a species identification guide describing the main characteristics of the commonly fished species, a spring balance and a measuring tape.

For each fishing trip, various items of information were to be recorded: date, main gear used (we made the hypothesis that they only used one type of gear during a fishing trip), whether they fished from a boat or from the shore, the travel duration (from home to the fishing site, and travel duration by boat if used), the fishing site (town and fishing sector, based on precise sectors on an attached map), the port of departure, the fishing duration, the description of the sea bass catches (weight, length, whether kept or not), and the description of the other catches (species common name, weight and number of both kept and released individuals). The panel methodology offers the possibility of obtaining precise information about released catches, which can generally not be inspected during on-site surveys [15], and about night fishing. 190 (74.2%) of the 256 fishers constituting the initial panel returned at least 1 fishing diary and 40 fishers (15.6%) returned the whole-year set of diaries, providing a seasonal picture of the fishery.

However, the sea bass-specific telephone survey combined with the panel study was inadequate for assessing the size of the recreational sea bass fishers population in whole France and the total French catches, since it only focused on the coastal departments. For extrapolating the results at a national scale, a pilot telephone survey, carried out in 2006 nationwide, was used. This second telephone survey aimed at providing the first global estimate of the French mainland recreational fishing (all species considered, including shellfish) by evaluating the total number of recreational fishers, the main target species and the related practices [35]. Two criteria of the nationwide telephone survey were considered for extrapolating the data of the sea bass-specific telephone survey: (1) having fished at least one time the last year in a coastal department included in the study area and (2) citing the sea bass among the three main targeted and fished species. Thus, using both the structure of the fishers' population living mainland (except in the coastal departments) and the respective weight of the fishers living in the coastal departments and in the rest of France, a crossing coefficient between fishers living in the coastal departments and fishers whom not was evaluated. By implementing this crossing coefficient, an estimation of the number of sea bass fishers living in the non-coastal departments was obtained (Table 1).

**Table 1:** Variables used for calculating the weighting factors and evaluated number of sea bass fishers in each stratum (coastal, non-coastal and whole France population).

Fishing frequency	Main fishing gear	Fishing mode	Coastal sea bass fishers population (2009 survey)	Non-coastal sea bass fishers population (estimation)	French sea bass fishers population (estimation)
Occasional	All	Shore	22,634	15,401	38,036
	All	Boat	21,450	7,006	28,455
Regular	Spearfishing	Shore	5,509	1,336	11,583
	Spearfishing	Boat	4,738		
	Lines	Shore	47,438	34,399	81,837
	Lines	Boat	44,523	58,023	102,546
Highly regular	Spearfishing	Shore	4,972	4,079	14,428
	Spearfishing	Boat	5,377		
	Lines	Shore	29,606	13,811	43,416
	Lines	Boat	39,004	10,682	49,687
Total fishers population			225,252	144,737	369,989

Occasional: fewer than 3 fishing trips/year targeting sea bass; Regular: between 3 and 15 fishing trips/year targeting sea bass; Highly regular: more than 15 fishing trips/year targeting sea bass. Population: number of coastal sea bass fishers in France, based on the national telephone interview.

### Weighting the data

To ensure the panel sample to be representative of the coastal and non-coastal French recreational sea bass fishers population, and to obtain reliable results at a national scale, the panel data was corrected using a set of weighting correction factors [35]. The calculation of the weighting factors was done applying the CALMAR program (http://www.insee.fr/fr/methodes/default.asp?page=outils/calmar/accueil_calmar.htm), used since 1990 by the French National Institute of Statistics and Economic Studies (INSEE) [36], [37].

The data was corrected by weighting the individuals, here the fishers. This was done using the values of some variables of adjustment, which were known both for the sample and the population. In our case, the considered variables used for calculating the weighting factors were the fishing frequency, the main fishing gear and the fishing mode (Table 1). Since the considered variables are category-based and since we knew the number of fishers belonging to each of these categories' modalities in the whole French population (2009 and 2006 telephone surveys, Table 1), the “ranking ratio method”, also named “iterative proportional fitting”, was used [38]. A weighting factor was attributed to each fisher for every month, and permitted to extrapolate the panel data to the whole French sea bass recreational fishers population.

### Data analysis

15,085 households were interviewed for the global telephone survey and 15,091 for the sea bass-specific one. A total of 134 (global survey) and 535 (sea bass-specific survey) sea bass fishing households were identified from these interviews. In 2009–2010, a total of 256 fishers agreed to be part of the panel. A total of 1,190 fishing trips and 1,383 sea bass catches were recorded from the 190 fishers who returned at least one fishing diary.

Fishing effort was equated with fishing duration, in hours. The CPUE (Catch Per Unit of Effort) was expressed as the total catch (in biomass [in g.] and/or abundance [in ind.]) per hour of fishing (g h^−1^ or ind h^−1^). The uncertainties of the catches estimations were assessed while analyzing the panel catches information per month, and calculating the precision of the information through the calculation of the mean, standard error and corresponding uncertainty of the sea bass catches information of the panel dataset. We found that over the year, the uncertainty could be evaluated at 51%.

The data analyses were performed using the R software [39]; all the results were standardized with the weighting factors using the “rgrs” – social sciences R-package.

### Robustness of the dataset

In order to evaluate the robustness of the panel declarations, the length-weight (L-W) relationship of the 1,383 sea bass caught by the volunteer fishers of the panel, measured by themselves and recorded in the diaries, was compared to the L-W relationship of 911 sea bass collected and measured by scientists between 2007 and 2011 during the EVHOE scientific campaigns conducted in the Bay of Biscay, English Channel and North Sea (Fisheries Information System Resource, IFREMER Brest) [40]. The panel catches covered a wide range of sea bass lengths: the smallest sea bass caught by the panel was 6 cm long, whereas it was 26 cm for the scientist dataset, and the largest sea bass caught by the volunteers was 82 cm long compared to 85 cm in the scientific dataset. The mean observed sea bass length was 38 cm in the panel dataset and 53 cm in the scientific set. The biggest declared sea bass caught by volunteers was of 6 kg while the biggest sea bass of the scientific survey was of 6.3 kg.

**Figure 3:**
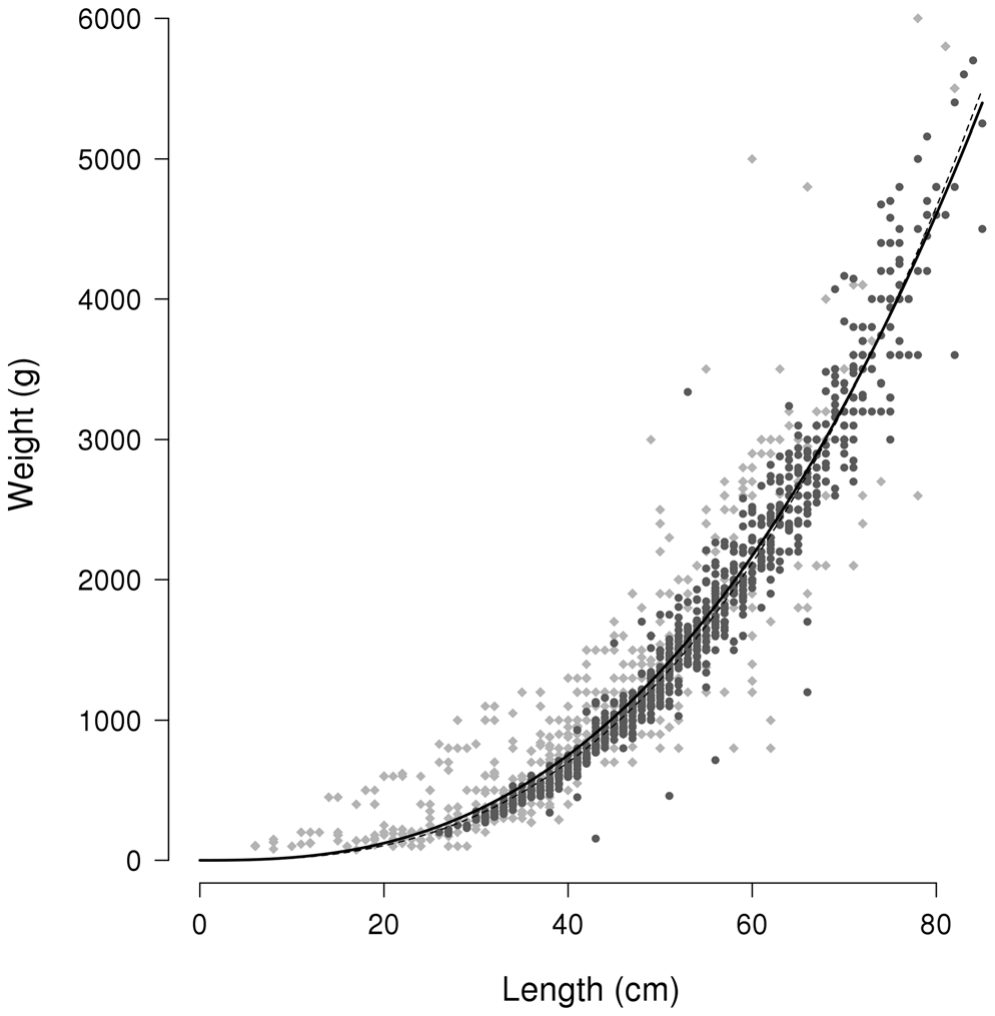
Length-weight (L-W) relationship of the European sea bass, based on the panel of volunteers and scientific catches. Dark grey circles: scientists' catches; Light grey squares: panel's catches; Solid line: L-W relationship based on scientists' dataset; Spotted line: L-W relationship based on panel's dataset.

The a and b parameters of the length-weight relationships were estimated using the “nls” non-linear least-squares function in R. The estimated L-W parameters of the panel dataset were a = 0.048536 and b = 2.615324 and those of the scientific dataset were a = 0.029122 and b = 2.734432. Although the “sm.ancova” statistical test of equality gave a p-value = 0, the regression of the panel dataset (bold solid line) and of the scientific one (dotted line) appeared extremely close (Fig. 3).

## Results

### Fishing gears

**Figure 4:**
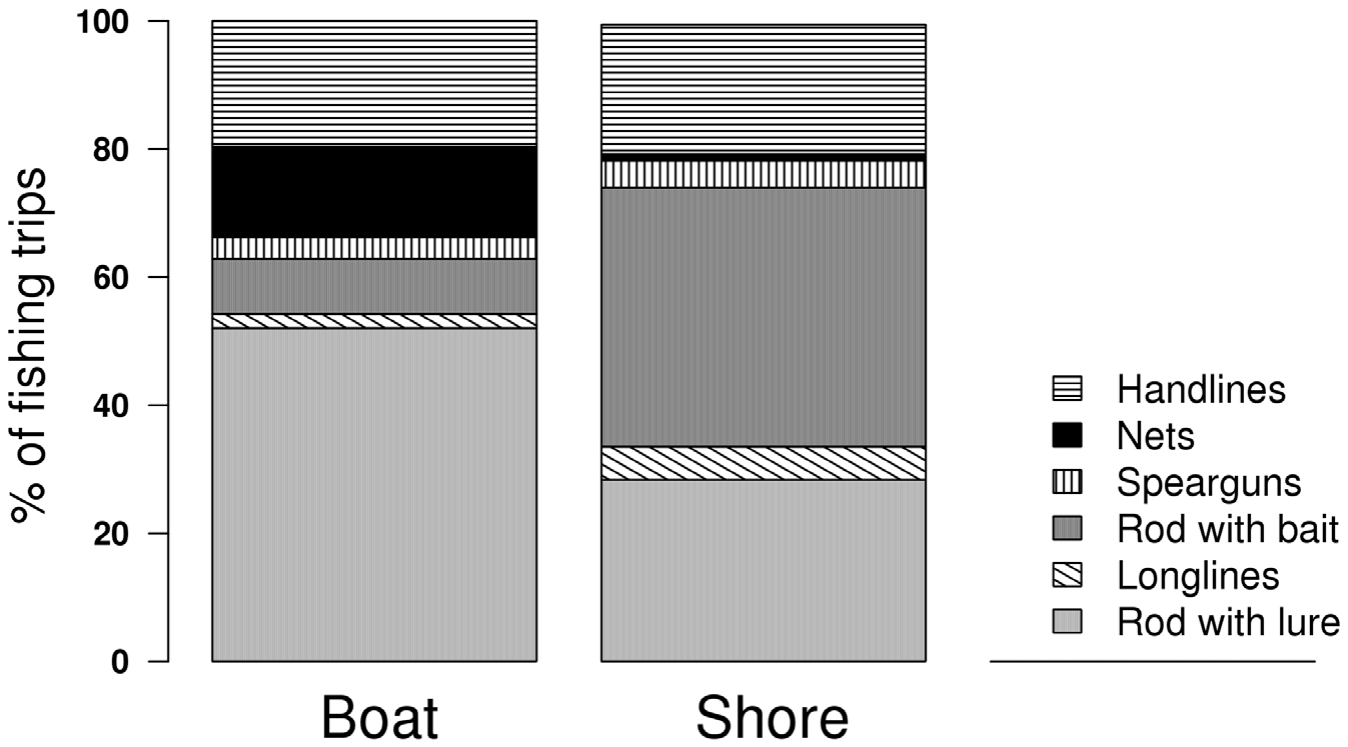
Percentage of fishing gear used for catching sea bass, from the shore and from boats.

Various types of gear were used for targeting the sea bass (Fig. 4). We recorded 7 of these: handlines, longlines, nets, spearguns, rod with bait, rod with lure, and fly-fishing rod. However, since only one member of the panel used this last type of fishing gear, it was not considered as representative of recreational fly-fishers as a whole, and was not considered.

The type of gear used depended on the fishing mode, meaning if the fisher fished from the shore or from a boat. The rod with bait was the mainly used type of gear from the shore, represented in 40.5% of the shore fishing trips while it was declared in only 8.6% of the boat fishing trips. However, the rod with lure was used in 52% of the boat fishing trips but only in 28.3% of the shore fishing events.

Longlines and spearguns were used by a small number of fishers. Both were used slightly more often from the shore (5.2% and 4.2% of the fishing trips, respectively) than from boats (2.2% and 3.3% of the fishing trips, respectively). Handlines were used as often from the shore as from boats, in about 20% of the reported fishing trips. Lastly, nets were mainly used from boats, representing 14% of the fishing trips but were only used in 1% of the fishing events displayed from the shore.

### Catch-and-release

The minimum legal catch size of the European sea bass in the Atlantic was 36 cm at the time of this study. The panelists' records showed that the mean length of caught sea bass was 38 cm, corresponding to a weight of 825.2 g. The mean length of the kept sea bass was 46.6 cm (1,230 g) while it was 29 cm (407.5 g) for the released ones.

**Figure 5:**
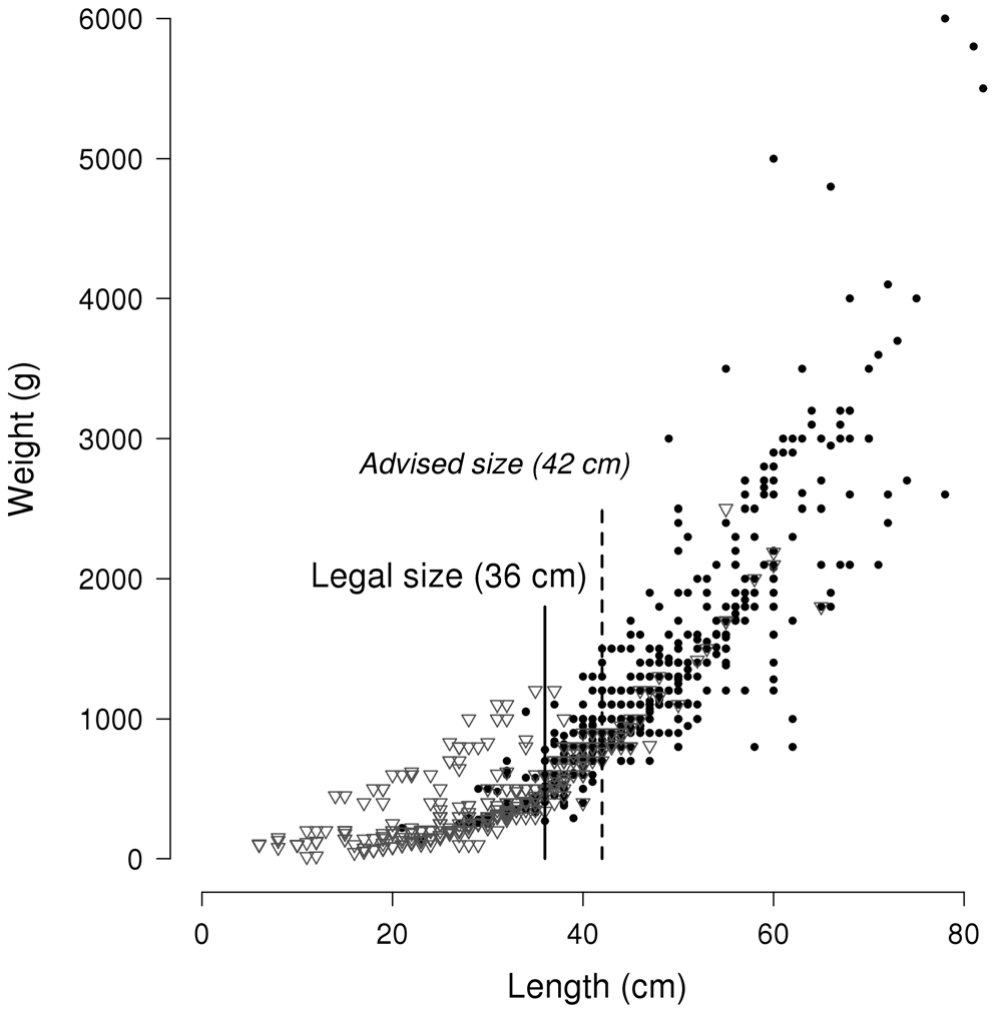
European sea bass kept and released by recreational fishers. Solid circle: kept sea bass; Filled triangle point down: released sea bass; Legal size: minimum legal catch length; Recommended size: length generally recommended by French recreational fishery federations.

When looking at the minimum legal catch size, it appeared that 87.9% of the undersized sea bass were released at sea, while 12.1% of them were kept (Fig. 5). However, 23.4% of the legal-size sea bass were also released at sea. Although 36 cm was, at the time of the study, the minimum legal size for the sea bass catches in the French Atlantic, recreational fishing federations generally recommended a minimum catch size of 42 cm, accounting for the reproductive characteristics of Atlantic sea bass stocks. It is to note that since the 26th of October 2012, the minimum legal size for the Atlantic sea bass recreational catches is 42 cm. Considering the caught sea bass having a size between the former legal one of 36 cm and the former recommended one of 42 cm, we observed that half of them were released at sea (48.6%).

### Catches characteristics per fishing trip

Analyzing the panel dataset, we found that a mean of 1.15 sea bass (or 953 g) were caught during a fishing trip; by mean, half of these catches were kept by the recreational fishers, and the other half released at sea.

**Figure 6:**
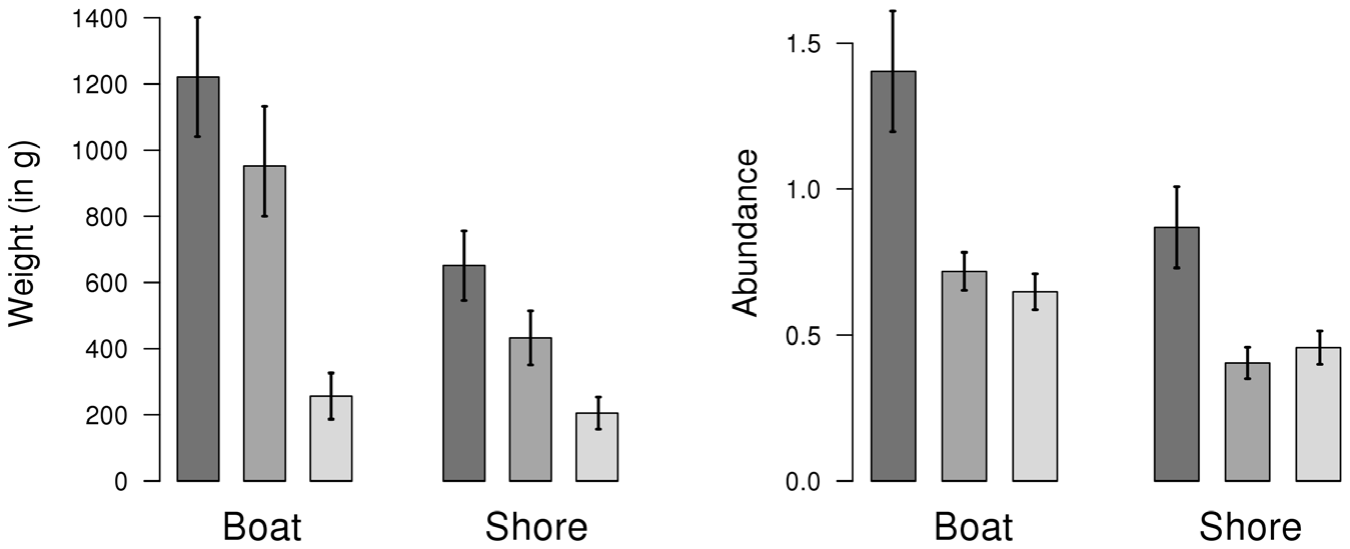
Mean weight and abundance of sea bass caught by recreational fishers from boats and from the shore. Dark grey: caught sea bass; Medium grey: kept sea bass; Light grey: released sea bass; Black bars: confidence interval (α = 0.95).

However, the mean amount of sea bass caught from the shore and from boats differed significantly both in biomass and abundance (Fig. 6): from boats, a mean of 1,221.1 g of sea bass (1.4 ind.) were caught per fishing trip, while 650.9 g (0.87 ind.) were caught from the shore. The biomass of released fish was much lower than that of kept fish, both when fished from boats (256.6 g released, i.e. 21% of the caught biomass) and from the shore (205.1 g released, i.e. 31.5% of the caught biomass). However, with respect to abundance, in each case about half of the caught sea bass were released at sea, that is 0.65 (46% of the catches) of those caught from boats and 0.46 (53% of the catches) of those caught from the shore.

**Figure 7:**
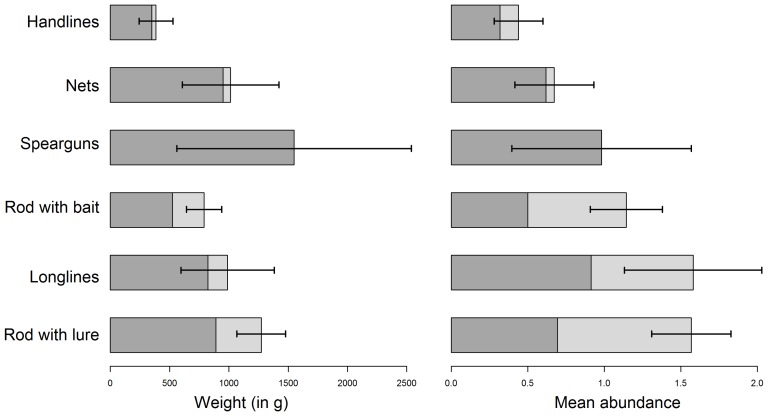
Mean weight and abundance of sea bass caught per gear type and fishing trip. Medium grey: kept sea bass; Light grey: released sea bass; Black bars: confidence interval (α = 0.95).

Recorded catches per type of gear also highlighted some particular patterns (Fig. 7). Spearfishers caught the highest biomass of sea bass per fishing trip (1,572.35 g) whereas the mean abundance was one of the lowest (1 ind.). All the spearfishing catches were kept. The highest abundance of sea bass caught per fishing trip were reported by fishers using rod with lure and longlines, each type reporting a mean value of 1.6 sea bass caught per fishing trip; however, the mean biomass was higher when using rod with lure (1,287.9 g) than longlines (991.8 g). The released amount of sea bass was 381.6 g (29.6%) for the rod with lure and 166.1 g (16.7%) for the longlines. With respect to abundance, fishers using the rod with lure released a mean of 0.87 sea bass per trip (54.4% of the catches) while those using longlines released a mean of 0.67 sea bass per trip (41.9% of the catches). Fishing with nets produced a fairly small number of sea bass catches per trip (0.71 ind.), but all or almost all of them were kept (87%). This resulted in a mean caught biomass of 1,037.1 g and a released one of 64.8 g (i.e. 6.25% of the catches). Handlines produced the lowest catches per fishing trip, both in biomass and abundance: 390.2 g were caught and 36.15 g released (9.3% of the catches), corresponding respectively to 0.45 individuals caught per trip among which 0.12 were released (26.7% of the catches).

### Catch Per Unit of Effort (CPUE)

The mean observed recreational fishing duration, all types of gear included, was 3h20 (Table 2). However, the mean and maximum durations varied depending on the type of gear. The two passive gears displayed the greatest mean fishing duration: nets were generally set for 5h30 (with a maximum of 24 h), and longlines were set for a mean of 4 h per fishing operation (max. 12 h). Rods with bait were reportedly used for a mean of 3h20 (max. 9 h), spearguns for 3h10m (max. 7 h), rod with lure for 3 h (max. 12 h) and handlines for 2h57 (max. 12 h).

**Table 2:** Mean, minimum and maximum observed fishing duration per type of gear, and number of fishing operations declared using the considered gear.

Gear	Mean	Min.	Max.	Nb of samples
Net	5h30	1 h	24 h	88
Longline	4 h	1 h	12 h	47
Fishing rod with bait	3h20	0h15	9 h	239
Fishing rod with lure	3 h	0h20	12 h	486
Handline	2h57	0h10	12 h	202
Speargun	3h10	0h15	7 h	49

**Figure 8:**
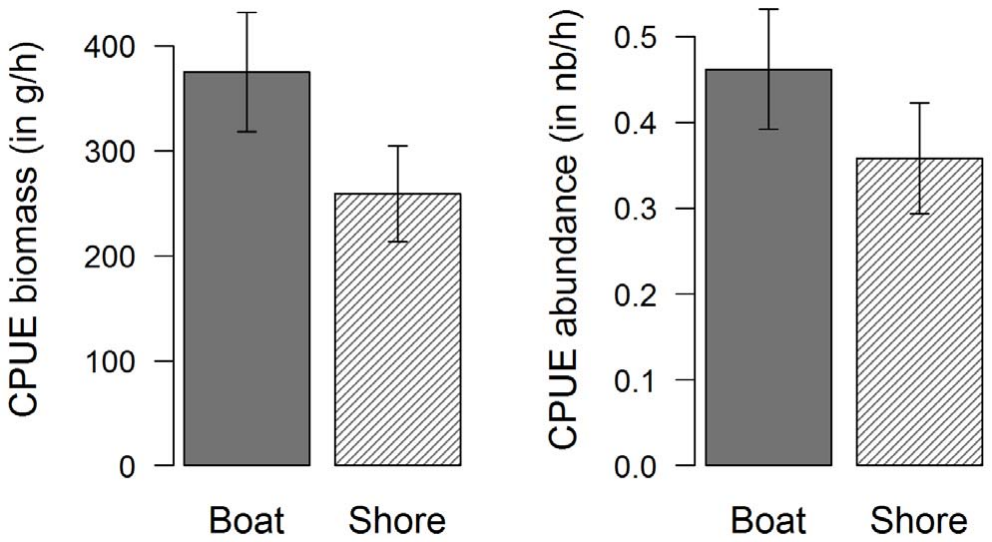
Mean CPUE of caught sea bass, from boats and from the shore. Dark grey: from boats; Shaded lines: from the shore; Black bars: confidence interval (α = 0.95).

In calculating the CPUE, we observed that a mean of 321.5 g h^−1^ of sea bass (corresponding to 0.41 ind h^−1^) were caught, all types of gear considered together. However, CPUE from boats were higher both in abundance and in biomass than CPUE from the shore (Fig. 8): 375.4 g h^−1^ and 0.46 ind h^−1^ were caught from boats compared to 259.2 g h^−1^ and 0.36 ind h^−1^ from the shore.

**Figure 9:**
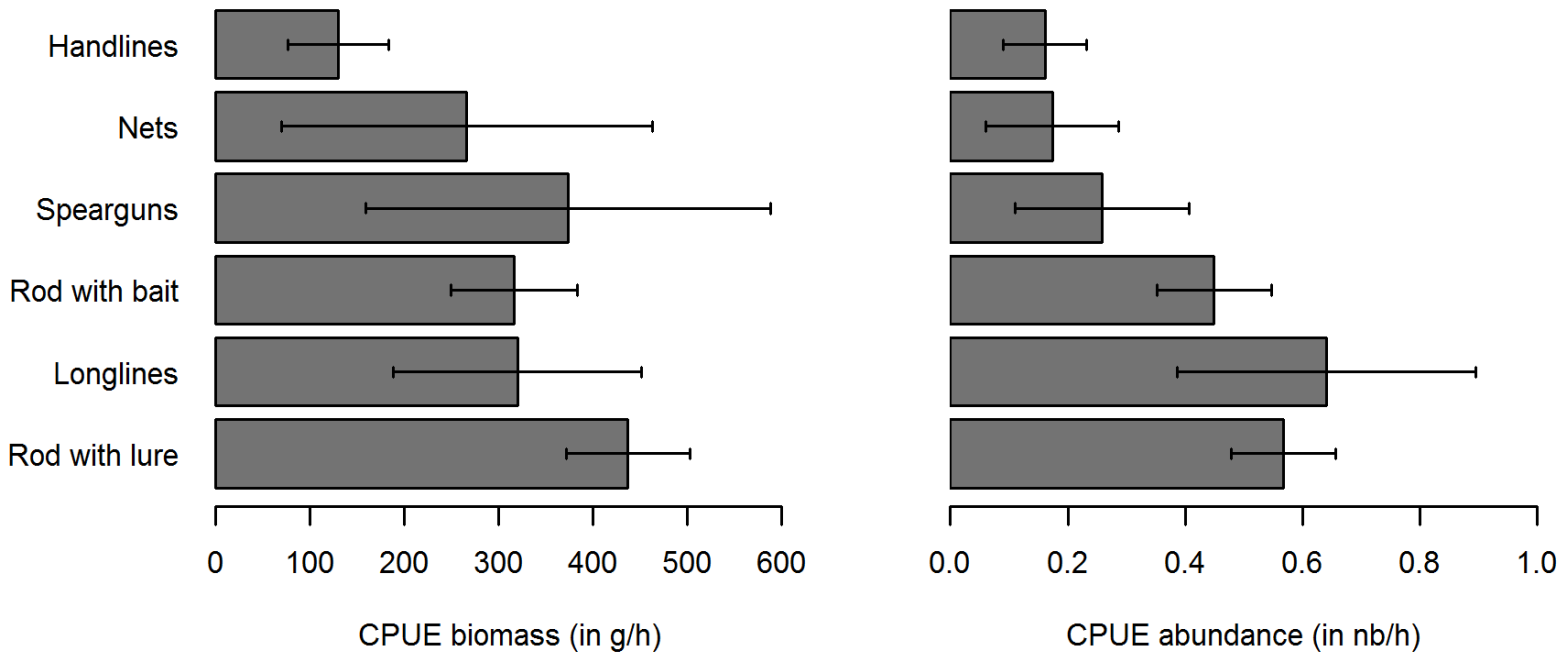
Mean CPUE of caught sea bass, per gear type. Black bars: confidence interval (α = 0.95).

We also observed differences when viewing the fishing gears separately (Fig. 9). With respect to biomass, rod with lure was the most effective gear, allowing to catch a mean of 437 g h^−1^ of sea bass. Handlines were least effective, with 130 g h^−1^ caught. With respect to abundance, longlines caught the greatest number of sea bass per hour of fishing, with 0.64 ind h^−1^, while handlines caught the lowest, with 0.16 ind h^−1^. Differences in CPUE by type of gear, comparing both biomass and abundance data, brought out some gear-dependent fishing patterns: both nets and spearguns displayed high values of CPUE in biomass (266.51 g h^−1^ and 373.8 g h^−1^, respectively) but low values of CPUE in abundance (0.17 ind h^−1^ and 0.26 ind h^−1^, respectively), indicating that mainly large individuals were caught, having a mean length of 53 cm and 50.6 cm respectively. In contrast, longlines had the highest CPUE in respect to abundance, with a mean of 0.64 ind h^−1^, but were in third place with respect to biomass, with a mean value of 320.3 g h^−1^, meaning that longlines mainly caught numerous but small sea bass, having an actual mean length of 36.2 cm.

**Figure 10:**
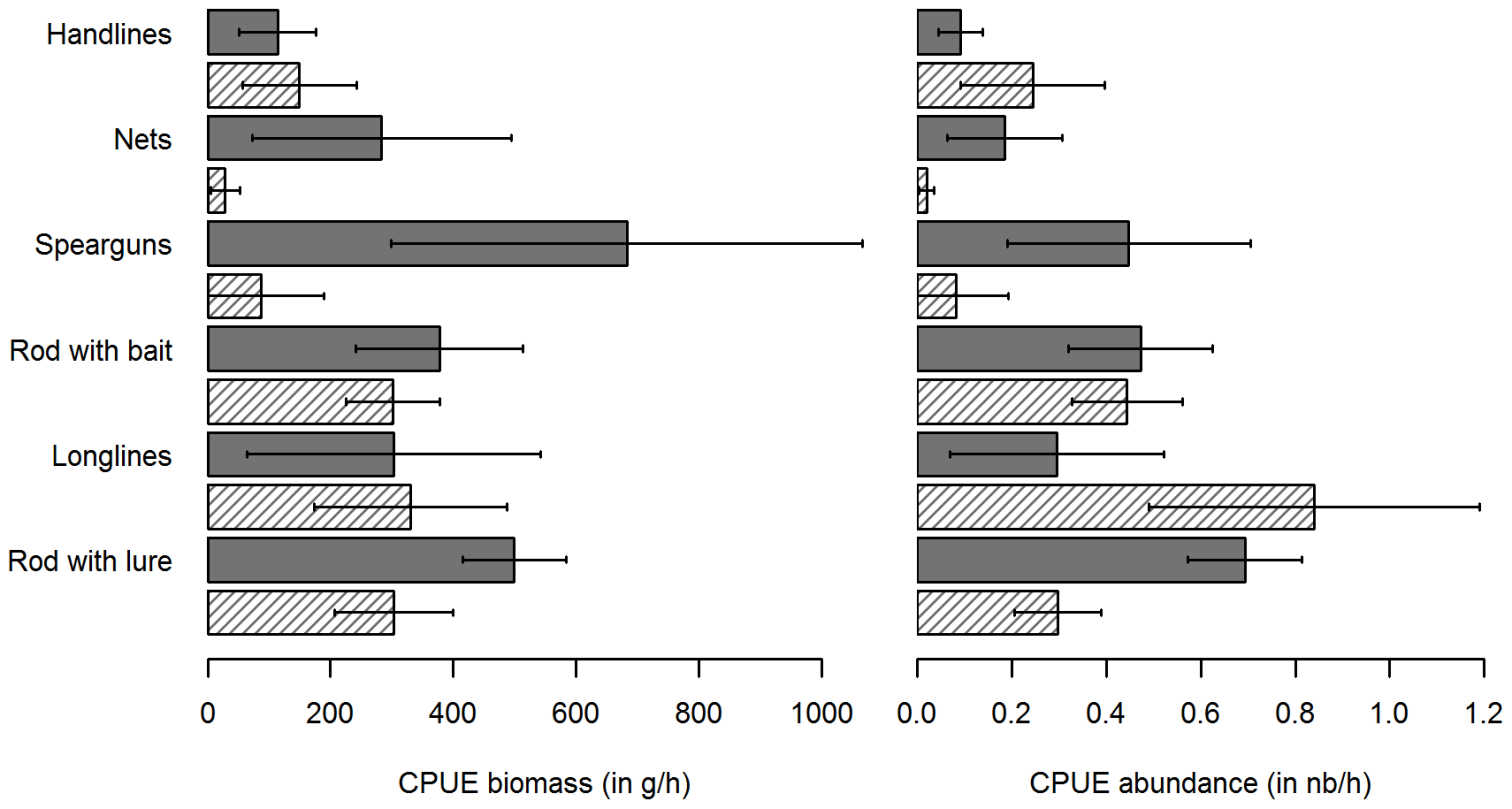
Mean CPUE of caught sea bass, per gear type and fishing mode. Dark grey: from boats; Shaded lines: from the shore; Black bars: confidence interval (α = 0.95).

Lastly, calculating the CPUE per fishing mode and type of gear brought out some additional differences (Fig. 10). For the majority of gear types, catches were observed to be greater when fishing from boats than from the shore, except in the case of longlines and handlines. However, the observed differences for these two gear types principally affected abundance and not biomass: in these cases, sea bass caught from the shore were smaller than those caught from boats. For spearfishing, differences were obvious both in biomass and abundance: there were more catches from boats than from the shore. The same pattern was also observed for nets: sea bass caught from boats were more numerous and the mean biomass was higher than when caught from the shore. However, these results must be viewed with caution since the dataset available for nets used onshore was relatively small (n = 7). The catches by rod with lure followed a different pattern: with respect to both abundance and biomass, while the mean CPUE in biomass was greater from boats than from the shore, this difference was even greater with respect to CPUE in abundance, indicating that more small individuals were caught from boats than from the shore. Lastly, CPUE observed when using a rod with bait did not differed when fishing from boats or from the shore.

### Total catches

After adjusting the panel data with the weighting factors issued from the two telephone surveys, the total number of recreational sea bass fishers in the Bay of Biscay, Channel and North Sea regions was estimated at 370,000 and the overall recreational sea bass fishing effort was estimated at 2,177,378 fishing trips per year in these areas. In terms of catches, it was estimated that 3,935,024 sea bass (±51%; min: 1,928,162 and max: 5,941,886) were caught by recreational fishers, of which 1,948,888 were kept and the others released at sea. In terms of biomass, this corresponded to a total annual catch of 3,173 tonnes (±51%; min: 1,554.8 t and max: 4,791.2 t), of which 2,345 tonnes were kept (74%). Coastal fishers caught two-thirds of this total (2,079 t, of which 1,546 t were kept) and fishers living inland caught the other third (1,094 t, of which 799 t were kept).

**Figure 11:**
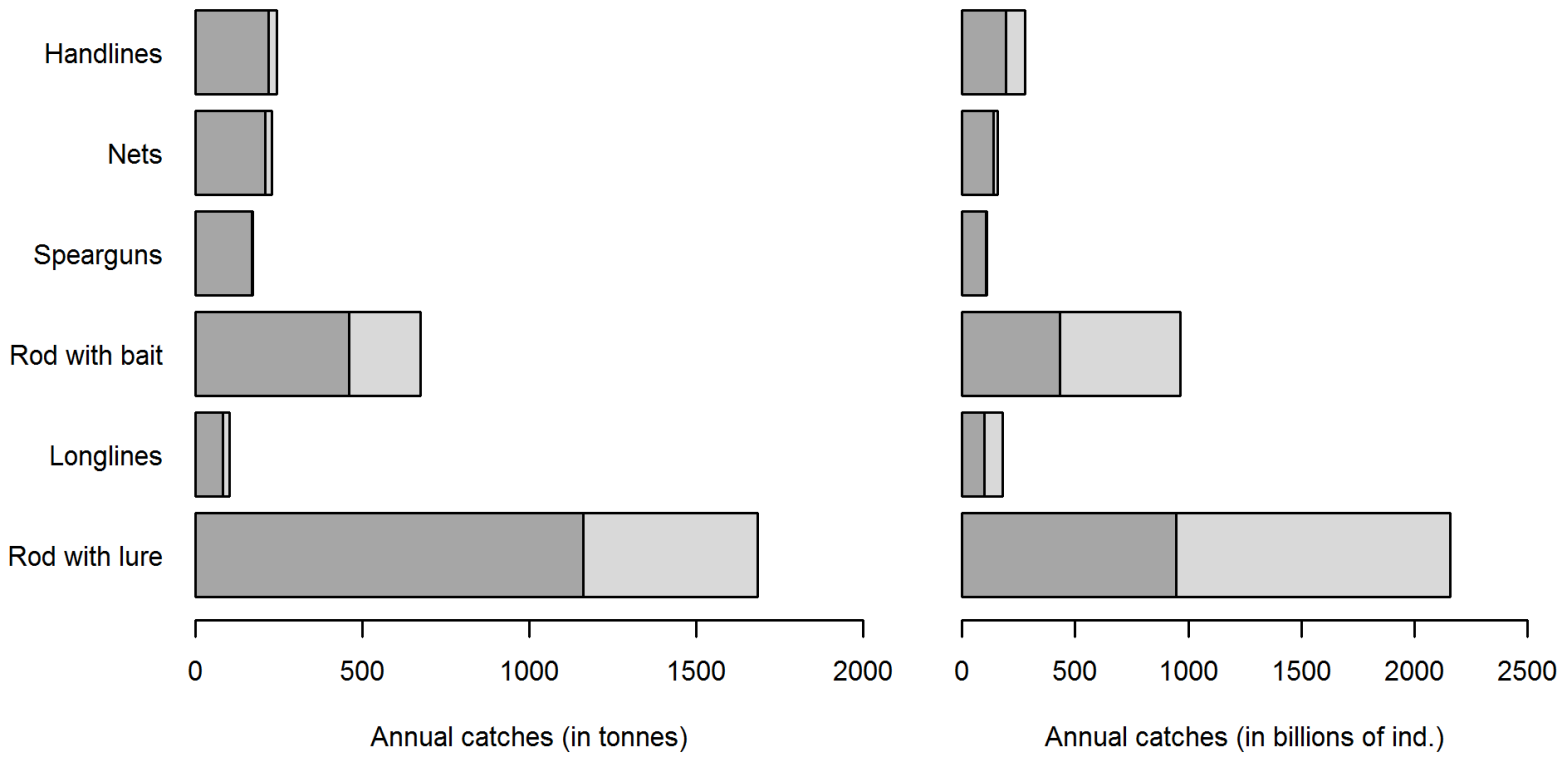
Annual recreational catch estimates of European sea bass in France (Atlantic coasts). Medium grey: kept amount; Light grey: released amount.

Total annual catches varied significantly depending on the type of gear used (Fig. 11). Rod with lure, rod with bait and handlines were the most often used gears in the reported fishing trips (representing 40.8%, 20.1% and 17% of the total reported trips, respectively). Fishers using rod with lure caught the largest proportion of sea bass annually, which was estimated at 1,683 tonnes (2.16 10^6^ ind.). Fishers using rod with bait caught 674.5 tonnes of sea bass (0.96 10^6^ ind.) in 2010. Fishers using other types of gear caught a smaller amount of sea bass, less than 300 tonnes per considered gear. Fishers using handlines, nets, spearguns and longlines caught the lowest amount of sea bass, that is 243.8 t (278.2 10^3^ ind.), 228.3 t (156.7 10^3^ ind.), 171.9 t (109.4 10^3^ ind.) and 101.1 t (179.2 10^3^ ind.), respectively. The total released biomass was observed to be around a third of the catches when using rod with lure and rod with bait (31% and 34% of the catches biomass, respectively). Fishers using other types of gear released a smaller percentage of the catches at sea: 8.4%, 10.5% and 17% of the sea bass biomass caught by nets, handlines and longlines, respectively. Since spearfishing generally results in the immediate death of the fish, no catch-and-release of sea bass was associated with this type of gear. The quantity of released fish was the highest for rod with lure (1.2 10^6^ ind., i.e. 56% of the catches) and rod with bait (530 10^3^ ind., i.e. 55% of the catches).

## Discussion and Conclusions

Even though various recreational fishing studies have been carried out in European and non-European countries (see [7], [18], [23], [24], [32], [33], [35], [41]–[48]), the lack of recreational and other non-commercial fishing data worldwide is still problematic [5], [49]. Thus recently, many countries in Europe, supported by the Data Collection Framework, have developed recreational fishery evaluations [24], [33]. Our study is the first to precisely describe and assess marine recreational fishing and catches of the European sea bass in France.

The nationwide telephone survey permitted to estimated at 2.5 million the number of marine recreational fishers in France (targeting all species including shellfish), representing 4% of the French population [35]. This can be compared to other countries records: in the Netherlands, it has also been estimated that 4% of the population are recreational fishers, fishing in marine coastal waters [50]; in Germany, including both inland freshwater and coastal marine waters, 4.7% of the population were considered as active anglers in 2002 [51], while the 2010 value, based on the sold fishing licenses, accounted 1,503,043 anglers, representing 1.8% of the German population [52]. In 2009, it was estimated that 137,000 anglers were fishing along the German Baltic Sea coast, representing only 0.17% of the population [33]. In contrast, in Australia the recreational fishing population has been estimated at 20% of the total population (80% of them fishing in marine and estuarine waters) [53]. Such a large number may be explained by the fact that 83% of the Australian population live within 50 km of the coastline. In summary, the proportion of the population involved in recreational fishing obviously varies substantially by country. Cooke and Cowx [6] have estimated that 11.5% of the world's population could be engaged in recreational fishing, but noted that this percentage can vary significantly according to the considered country.

### The sea bass recreational fishery

We found that the mean weight of a sea bass caught from a boat (1.22 kg of sea bass caught and 0.96 kg kept per trip) was twice that when caught from the shore (0.65 kg caught and 0.45 kg kept). There was also a large difference in abundance: a mean of 1.4 sea bass was caught from boats compared to 0.87 from the shore. These differences between boat and shore catches have already been observed in other recreational fishing studies in the Atlantic and the Mediterranean, and also in freshwater areas. Pickett and Pawson [12] estimated that 0.94 kg of sea bass were retained by marine recreational fishers in the UK when fishing from boats and 0.8 kg when fishing from the shore. They also showed that a higher proportion of sea bass caught from the shore was released compared to that caught from boats, suggesting that shore recreational fishers usually catch smaller bass. In the Mediterranean, the same pattern has also been observed, since recreational fishing catches from boats were higher than ones from the shore [45].

The mean catch length varied according to the gear type. Our study showed that on the French Atlantic coasts, sea bass were targeted by recreational fishers using a wide variety of gears. This was also observed in recreational fisheries of other European countries like in Denmark, UK, Germany and Sweden [24]. We noticed that the type of gear used depended on the fishing mode: fishers fishing from a boat mainly used the rod with lure while those fishing from the shore mainly used the rod with bait. These observations could be explained owing to the fact that on one hand, bait used for catching sea bass, like sand eels or shrimp, can be easily found by shore anglers on the fishing site itself. On the other hand, there are various ways to catch sea bass with lure from a boat, like among others the troll, the spinning or the vertical fishing, whereas from the coast, the main used technique is the spinning. Thus, the possibilities to use lures from a boat are broader than from the coast.

These differences can help to explain the observed CPUE trends. The smallest sea bass were caught using longlines, which were used more often from the shore than from boats, which can signify that small sea bass are more numerous along the shore. In fact, additional information on sea bass ecological niches indicated that young fish mainly live near the shore and tend to move to deeper waters as they get older [12], [54]. However, longlines have a great number of hooks, increasing the likelihood of catching several fish at once. That is why the CPUE of longlines used from shore was higher than that from boats, indicating larger catches mainly composed of small individuals.

The largest sea bass were caught by recreational fishers using nets. This gear was mainly used from boats, meaning that the fish could be pursued in deep water where the largest sea bass typically live. Nets may also be used primarily by fishers having a greater experience in fishing for sea bass, who may know more about where and how to catch larger fish.

Spearfishing is generally viewed as a highly selective fishing method [45]. Indeed, we observed in this study that the mean catch length was 50 cm, much larger than the minimum legal size, 36 cm, and the recommended size of 42 cm. With respect to CPUE relative to biomass, rod with lure and spearguns were the most effective gears, but spearfishing induced low CPUE relative to abundance, indicating that the targeted sea bass were mainly large ones. Spearfishers generally target fish considering their emblematic value, their taste, their ease of catching and their length [55]. Since in spearfishing there is no possibility of catch-and-release, and that all caught fish are kept, spearfishers generally target the largest ones, both to comply with the minimum legal catch length and for their trophy value.

### Catch-and-release

Catch-and-release is known to be a common practice in recreational fisheries [10], [56]–[61], as many people fish for fun and not mainly for their own consumption. Our study showed that this practice is prevalent in recreational sea bass fishing in France, since half of the caught sea bass were released. An equivalent release proportion of caught and released fish from recreational fishing has also been observed in various European countries [62] as well as in the US, with an estimate of 57% of released fish [10]. However, it is important to note that the released proportion of the catches was always greater in abundance than in biomass, showing that released sea bass catches were mainly composed of small fish.

Recreational fishers who keep the biggest fish while releasing the smallest ones can be seen as behaving responsibly, since catch-and-release is generally viewed as a conservation approach [61], [63]–[65]. However, although it decreases direct mortality from recreational fishing, this must be set off against the potential lethal consequences of catch-and-release [66]. Indeed, various outcomes of catch-and-release have been demonstrated to have direct and/or indirect effects not only on the survival of the caught fish but also on the population structure [67]. Deep hooking, depending on factors such as type of bait, hook size and gear configuration can negatively impact the fish's survival [68], [69]. The stress endured by the fish during the catch-and-release events can induce physiological and behavioral alterations (like increased vulnerability to predation) which can lead to the death of the fish [67]. Fish mortality after catch-and-release is an essential element to evaluate for adapting fishing regulations; however, this is highly dependent on variables such as the considered species, the gear used [10] or the seawater temperature [70].

### Commercial and recreational sea bass fisheries

The European sea bass is an important species, both for recreational and commercial fisheries. In the UK, it was yet targeted by anglers since the early 19th century, whom regarded this species as a gamefish, but was already known as a valuable food fish in the Mediterranean, particularly in France, Spain and Italy [12]. However, the interest of commercial fisheries is more recent, beginning to specifically target sea bass in the late 1970's [12], [25]. Commercial sea bass fishing is divided into inshore fishing with small boats using a great variety of gears and displaying little activity during the winter, and offshore fishing, where mid-water pair-trawlers target pre-spawning and spawning sea bass during the spawning season between November and April [25], [71].

We have estimated that recreational sea bass fishers of the Bay of Biscay, the Channel and the North Sea caught a total of 3,174 tonnes of sea bass and landed 2,345.5 tonnes per year, in other words a mean of 8.6 kg of sea bass caught and 6.3 kg kept per fisher per year. In the same areas and at the same time, landings by French commercial fishers have been estimated at 5,160 tonnes [71]. Thus, the total sea bass landings on the western coasts of France amounted to 7,505 tonnes, with recreational fishing accounting for around 30% of total landings.

Other studies have also shown that recreational fishing can represent a significant part of total landings [6], according to the considered species and area. In some cases, recreational fisheries are known to catch more fish than their commercial counterparts [4], like for the high-quality dolphin-fish (*Coryphaena hippurus*), targeted in the United States [72], and the lingcod (*Ophiodon elongatus*), where recreational landings account for 60% of total catches [15]. It has also been observed in the US that recreational landings of the Atlantic striped bass (*Morone saxatilis*), an emblematic species for recreational fishers, exceed those of commercial landings, and account for 74% of total landings [10]. In the Mediterranean, a global study estimated that leisure catches represent 10% of total landings [9]. In more specific areas, higher values can be reached, as observed in Cape Creus (Spain), where recreational fishing, while submitted to specific regulations and restrictions, is estimated to account for 30% of the commercial artisanal fishery production in the marine protected area [27], and in Mallorca, where recreational fishing production accounts for 27.5% of the commercial one [45].

### Sampling design and data quality

The sampling design we used for the telephone surveys did not allow perfect coverage of the whole target population of marine sea bass recreational fishers. Indeed, for the two telephone surveys, a database of registered phone subscribers was used. However, this database did not record all the existing French phone numbers. It is known that some landline telephone numbers were registered in a red list and were thus not available in the used national telephone directory. This part of the population, whom decided to hide their phone number, has been yet studied by the BVA polling institute, and it has been evaluated that they well represent the rest of the subscribers (BVA, personal communication). Then, this missing part of the population should not induce bias in the results extrapolation. For the mobile-only group (people having only a cell phone and not a landline phone number), the subscribers could also decide not to be registered in the telephone directory. This represents ca. 15% of these subscribers; however, for these ones, we did not dispose of any information concerning their similarity to the rest of the population and at this point, we were not able to evaluate its associated bias.

The panel was constituted by fishers randomly called but whom deliberately decided to fill in fishing diaries. Generally, fishers who agreed to give their data are known to be the most involved in fishing and the most avid ones [52], [73]–[77]. In such case, if it is not possible to evaluate this bias, the obtained total results would be biased upward. To counteract this phenomenon, we associated all fishers to 10 different strata depending on 3 categories of variables, available at both the panel and the population level (evaluated during the telephone surveys): the main gear used, the fishing mode and the fishing frequency. Increasing the number of variables and/or modalities would permit the reduction of the bias, but this would be possible only with a greater number of panelists, to have samples big enough to properly represent each stratum. The weighting factor attributed to each panelist fisher permitted to counteract the over representation of some stratum on the panel, regarding the whole fishers population [78]. Since the panelists were recruited during the second telephone interview, covering only the coastal departments, we did not dispose of fishing diaries filled in by non-coastal fishers, generally most occasional ones, fishing during holidays. Thus, a correction of the panelist records was done by the BVA polling institute whom evaluated the representativeness of the non-coastal fishers regarding the coastal ones.

Non-response as well as respondent refusal generate unknown bias in the sample estimates and the results extrapolation [79]. The non-response problematic can be induced by a limited coverage of a study or by the voluntary non-response of the interviewed persons. The non-response issue was thus present at various steps of our study, including the telephone surveys and the fishing diaries one. We highlighted various levels of non-response: (1) no phone answer (absence or no phone), representing a total of 59% of the exploited phone numbers; (2) persons who refused to answer, here 29% of the called numbers; (3) panelists who did not return all the fishing diaries (79% of the panelists whom returned at least one fishing diary), (4) panelists not declaring some fishing trips and (5) panelists not declaring all of the catches. In our study, 172,054 calls were done for obtaining 15,091 complete interviews (9%). Thus, 91% of the calls were not effective, due to various reasons (absence, refusal, occupied, fax/modem number, already contacted…). The persons who refused to answer could then represent a strong source of bias. The main problematic of the non-response is to evaluate if the non-respondents differ from the respondents [80]. Concerning the no-phone answer, it is particularly complicated to know if the non-respondents (here 59% of the generated phone numbers) were similar to the respondents. However, for limiting the bias associated with this type of non-response, the polling institute recalled all the generated phone numbers up to 12 times, at different days and hours. This sampling design permitted to assume that each household had the same probability to be contacted by the polling institute, and consequently that the respondent group was similar to the non-respondent one. However, 29% of the contacted households directly refused to answer the telephone interview, and any complementary survey was available to verify if this non-respondent group was similar to the respondent one. It was thus impossible to evaluate the bias induced by these non-respondents.

At the next step, we observed that 12.7% of the households with a sea bass recreational fisher refused to continue the phone interview, inducing a response rate of 87.3% at this step of the study. This rate is generally considered good enough to offer a good representation of the target population, inducing low risks of bias [80], [81]. However, since some socio-demographic variables of the recreational fisher refusing to continue the interview were known, like the gender, the age and the activity, it was therefore possible to identify the category of the non-respondents and to compare it to the respondent ones for correcting the data.

Not all the panelists returned the fishing diaries for the whole year. Indeed, 190 of them returned at least one, and only 40 of them returned all the diaries. For limiting the bias induced by the fishers non-response during the fishing diary survey and to compensate for incomplete data, the applied weighting correction factor was fisher- and month-specific. The weighted component was not the individual but the fishing trips, which were monthly-representative. Thus it was not considered as problematic if some recreational fishers decided to stop the panel participation after returning the first, second or third fishing diary.

For the fishing diary survey, there was a risk that the fishers did not declare all their fishing trips neither all their catches. They could for example not declare an unsuccessful fishing trip, where any fish was caught, which could lead to an overestimation of the total catches, while missing the “zero-data”. They could also be tempted to hide a high rate of catches and to not declare some of them, such as the kept undersized fish, which in this case would lead to a final underestimation of the total catches. For evaluating the importance of such bias, it has been proposed to 47 panelists to be contacted every week during 3 months to fill in the fishing diary with them. The objective was to compare the fishing diary information obtained with the fishers who filled in the diary alone and those when the fishers filled in the diary with help and intensive reminder. A group of 14 panelists (30%) agreed to be regularly followed by the polling institute. The socio-demographic and avidity profiles of the panelists who agreed to participate were the same as those of the non-participant (33 recreational fishers). Even if the success of returned diaries was higher for the followed participants, the mean number of trips recorded was not very different (5.2 fishing trips/week for the followed fishers and 4.9 fishing trips/week for the non-followed ones), neither was the mean number of sea bass caught per fishing trip (1.98 for the followed fishers vs. 1.89 for the non-followed fishers). Even if more diaries were returned by the fishers contacted weekly, the low frequency of contact did not seem to be a great source of response bias. Whereas the number of followed fishers was low, these results gave a preliminary information concerning the reliability of the data voluntarily provided by the fishers themselves.

### Limitations

This study presents the first estimates for the recreational sea bass fishery on the Atlantic French coasts. However, although it gives much important information, other data are still needed to evaluate the entire French recreational sea bass fishery. This study did not account for the Mediterranean production, since it was designed to monitor sea bass catches requested by the DCF, which was limited to the Bay of Biscay, English Channel and North Sea. It also lacked reliable data concerning fly-fishing: only one fly-fisher agreed to be part of the panel and described only two fishing trips, which could not be considered as representative of this type of fishing activity. Thus, the annual amount of sea bass caught by fly-fishers, here estimated to be 5.2 tonnes, is still to be determined. Moreover, sport fishing, in the sense of official fishing competitions, as well as commercial angling charters were not taken into account in this study, since we did not dispose of any indicative estimates concerning their dimension. Finally, as already noted, catch estimates did not take into account the sea bass catch-and-release mortality [15], which is still unknown.

We used CPUE accounting for the fishing duration as the unit of effort, and compared various gears CPUE, while not accounting for the number of hooks or the length of the nets. Indeed, we aimed to compare the catches between different categories of fishers and not to compare the effectiveness of the gears themselves. However, comparing the CPUE of different gears, using for example the number of hooks as the unit of effort, would enhance the knowledge of the sea bass fishing techniques and characteristics. Since we did not dispose of such information in this study, it was not possible to evaluate these characteristics.

Methodologically, we defined a sea bass fisher as a fisher having caught at least one sea bass during the year. In other studies the only intention of catching sea bass was taken into account, and such fishers were also treated as sea bass fishers [12]. Our approach may thus have led us to underestimate the real number of recreational sea bass fishers.

Volunteer investment in scientific research can have some limitations that are important to point out. The panel survey may present some risks of bias by fishers who provided catch information. We have observed a tendency to round off, with many fish reported at exactly 40, 50, or 60 cm. This behavior has already been observed in other studies, and it seems that rounding errors are common [15]. This may be due to the fact that measuring a living fish is not easy before its release. However, we have seen that the length-weight relationship calculated on the panel dataset was very close to that of scientific estimates. The panel data can thus be viewed as of high quality despite these rounding errors.

Levrel et al. [82] have proposed three types of parameters which might influence the quality of volunteer-derived information: the availability of survey guidelines, the validation of datasets, and the ability to coordinate a network that includes different communities of practice. With respect to the first parameter, the polling institute directly contacted the volunteer fishers by telephone to explain the study design. The most important points were noted in the fishing diary supplied to each fisher, and a guide for fish identification was also sent out. Secondly, the validation of the dataset was managed by both the polling institute and the research team, while verifying all the parameters of the fishing trips and the catches amount, checking for extreme values or typing errors. Thirdly, recreational fishers were contacted by the polling institute every three months to get informed on the progress of the study, to answer their questions and to motivate the panelists to continue. The fishers also had direct phone access to the scientist in charge of the study, and were encouraged to phone at any time if they faced any problems.

Despite the possible limitations of a volunteer-based large-scale study, such a participative methodology has many advantages: it can help to secure the participation and cooperation of recreational fishers in management decisions [83]; it is cost-effective [82]; working in collaboration with recreational fishers is a good way to obtain data otherwise hard to collect and to foster the participation of fishers in recreational fisheries management [15], [27]; and using volunteer self-declarations also makes it possible to collect information on discards [83], which is more important than ever but has been until now a gap in many catch estimates [3], [84], [85].

### Implications for management

Our study gave new information and highlighted the importance of the sea bass recreational marine fishery in the French littoral of the Bay of Biscay, English Channel and North Sea. We have underlined that this concerns 370,000 fishers, that around 4 million of sea bass were caught, and half of them were kept, representing 30% of the total catches including commercial landings. Such values are not anecdotal, and the question of the impacts of these catches on the stocks, not taken into account until this time, should be considered.

Some regulations for recreational fisheries in general, which hence concerns the sea bass, are yet implemented: The ministry of Ecology, Sustainable Development and Energy, as part of the “Grenelle de la mer” (2009–2012), which was a public process for reflection and negotiation between the State, the elected representatives, the economic and professional stakeholders concerned by the sea and the civil society, has developed some documents and actions concerning marine recreational fishing. On July 7th 2010, a charter of commitments and objectives for ecologically responsible marine recreational fisheries (http://www.developpement-durable.gouv.fr/IMG/pdf/CHARTE_peche_maritime_de_loisir_eco-responsable_signee_-2.pdf) was signed. On the 1st of July 2012, the Ministry opened a website for the online declaration of marine recreational catches (http://www.developpement-durable.gouv.fr/Declarez-pechez.html), but such declaration is still not obligatory. Moreover, a decree was established on May 17th 2011, requiring all recreational fishers to mark the catches they keep by cutting the lower part of the caudal fin (http://www.legifrance.gouv.fr/jopdf/common/jo_pdf.jsp?numJO=0&dateJO=20110527&numTexte=45&pageDebut=09187&pageFin=09188), in order to discourage the illegal sale of recreational catches. Moreover, the minimum catch length for the sea bass caught by recreational fishers fishing in the French Atlantic waters was raised from 36 cm to 42 cm on October 26th 2012, with the support of the recreational fishing federations.

Further management measures, concerning both commercial and recreational fishing of the sea bass, may be created later. Indeed, the ICES working group on Assessment of New MoU (Memorandum of Understanding) Species (WGNEW), created in 2005, aims to collect information on total international landings and research vessels survey data to evaluate stocks abundance trends, among which the sea bass stocks. The results of this working group are provided to the others ICES working groups WGHMM (Working Group on the Assessment of Southern Shelf Stocks of Hake, Monk and Megrim, for the Bay of Biscay Ecoregion) and WGCSE (Working Group for the Celtic Seas Ecoregion), in charge of modelling the stock evolution and proposing further recommendations if necessary. The 2013 ICES advices for the European sea bass are now available for the Bay of Biscay (http://www.ices.dk/sites/pub/Publication%20Reports/Advice/2013/2013/Bss-8ab.pdf) and for the English Channel and North Sea (http://www.ices.dk/sites/pub/Publication%20Reports/Advice/2013/2013/bss-47.pdf). For both regions, according to the ICES approach to data limited stock (DLS), a decrease of respectively 20% and 36% of the commercial landings is recommended. However, whereas the results of our study were presented and considered in these advices, recreational catches were still not taken into account by the ICES committee for the annual evaluations and recommendations, mainly owing to a lack of historical dataset.

Closer to the publication of these advices, the European Union has asked that each Member State recommend various management propositions for the European sea bass. All the possibilities were explored, concerning both commercial and recreational fisheries, but at this time, any official documents are available.

However, since we have developed another large-scale study on the European sea bass (among other species) recreational fishing in 2011–2012, including also the Mediterranean Sea, we hope that the new results, giving additional information and providing a second value of recreational fishing, will contribute to improve the management of the sea bass commercial and recreational fishery.
